# Extracellular association of APP and tau fibrils induces intracellular aggregate formation of tau

**DOI:** 10.1007/s00401-015-1415-2

**Published:** 2015-04-14

**Authors:** Muneaki Takahashi, Haruka Miyata, Fuyuki Kametani, Takashi Nonaka, Haruhiko Akiyama, Shin-ichi Hisanaga, Masato Hasegawa

**Affiliations:** Department of Dementia and Higher Brain Function, Tokyo Metropolitan Institute of Medical Science, Setagaya-ku, Tokyo, 156-8506 Japan; Department of Biological Science, Laboratory of Molecular Neuroscience, Tokyo Metropolitan University, Hachioji, Tokyo 192-0397 Japan

**Keywords:** Tau, Amyloid precursor protein, Propagation, Alzheimer’s disease, Down’s syndrome

## Abstract

Alzheimer’s disease (AD) is characterized by extracellular amyloid β (Aβ) deposition and intracellular tau aggregation. Many studies have indicated some association between these processes, but it remains unknown how the two pathologies are linked. In this study, we investigated whether expression of amyloid precursor protein (APP) influences extracellular seed-dependent intracellular tau accumulation in cultured cells. Treatment of tau-expressing SH-SY5Y cells with Aβ fibrils did not induce intracellular tau aggregation. On the other hand, in cells expressing both tau and APP, treatment with tau fibrils or Sarkosyl-insoluble tau from AD brains induced intracellular tau aggregation. The seed-dependent intracellular tau aggregation was not induced by expression of APP lacking the extracellular domain. The amount of phosphorylated tau aggregates in cultured cells was dose dependently elevated in response to increased levels of APP on the cell membrane. Our results indicate that the extracellular region of APP is involved in uptake of tau fibrils into cells, raising the possibility that APP, but not Aβ, influences cell-to-cell spreading of tau pathologies in AD by serving as a receptor of abnormal tau aggregates.

**Electronic supplementary material:**

The online version of this article (doi:10.1007/s00401-015-1415-2) contains supplementary material, which is available to authorized users.

## Introduction

Alzheimer’s disease (AD) is a progressive neurodegenerative disorder, characterized by the deposition of two kinds of filamentous aggregates: extracellular senile plaque consisting of amyloid β (Aβ) and intracellular paired helical filaments consisting of tau proteins. Aβ is produced by β-secretase- and γ-secretase-mediated cleavage of amyloid precursor protein (APP). The APP gene is located on chromosome 21, and trisomy of this chromosome is associated with Down’s syndrome, which exhibits an AD-like pathology. Furthermore, several missense mutations of the APP gene at or near the cleavage sites cause Aβ production in familial forms of AD [4, 11, 40, 41]. Consequently, abnormal production and deposition of neurotoxic Aβ were proposed to be the primary cause of AD: the amyloid cascade hypothesis [53]. Tau protein is one of the microtubule-associated proteins that stabilize microtubules and promote their assembly [23]. Six tau isoforms are expressed by alternative splicing of the mRNA in adult human brain, but in AD, all these tau isoforms are accumulated in hyperphosphorylated and partially ubiquitinated forms as a unique paired helical filament (PHF) structure in neurofibrillary tangles and threads. Both Aβ and tau fibrils in AD brains have a cross-β structure similar to that of abnormal prion protein in Creutzfeldt–Jakob disease [20]. The distribution and spreading of abnormal tau pathology in AD are temporally and topologically stereotypical, and correlate with clinical phenotype [1].

Based on the amyloid cascade hypothesis, considerable effort has been invested in understanding the relationship between Aβ and tau. Many studies have found some association between these proteins; for example, cytotoxic Aβ induces phosphorylation of tau [3, 7, 22, 33]. However, it remains unclear how they are associated. Vaccination with Aβ results in clearance of amyloid plaques in patients with AD, but fails to improve clinical symptoms or tau pathologies [10, 29, 34], suggesting that inhibition of Aβ formation or increased clearance of Aβ may not influence tau pathologies. In addition, Braak et al. [2] reported that tau aggregation precedes diffuse plaque deposition, and they hypothesized that Aβ may be released from non-junctional varicosities of axons generated from abnormal tau-containing brainstem nuclei in sporadic AD.

Recent studies using cellular and animal models have suggested that intracellular abnormal protein pathologies, including tauopathy, spread by a prion-like mechanism [9, 42]. Namely, an abnormal form of these proteins once formed in cells promotes the conversion of monomeric normal protein to an abnormal form. The abnormal proteins are also transmitted from cell to cell, resulting in spreading of the pathologies. Prion-like propagation of abnormal tau and α-synuclein has been demonstrated in transgenic and non-transgenic wild-type mouse models by direct inoculation into mouse brains of fibrils made of recombinant proteins and abnormal proteins from brains of patients [5, 6, 19, 31, 32, 35, 39].

Propagation of these proteins has been proposed to occur through various mechanisms, including indirect transmission by exocytosis and endocytosis, and direct transmission via nanotubes [8, 12, 27, 37]. However, nothing is known about the molecular mechanisms by which these intracellular abnormal proteins are secreted from cells and incorporated into other cells, and whether or not incorporation of these extracellular proteins is receptor mediated.

In this study, we investigated whether Aβ or its precursor protein APP is involved in incorporation and propagation of intracellular abnormal proteins. We show that overexpression of APP accelerated extracellular seed-dependent aggregation of tau, and also that APP-expressing cells treated with tau fibrils exhibited induction of Sarkosyl-insoluble tau aggregates in the absence of any transfection reagent. In contrast, overexpression of APP lacking the extracellular domain or treatment with Aβ did not accelerate tau aggregation. These results suggest that the extracellular domain of APP is involved in the incorporation of tau fibrils into cells; in other words, APP may serve as a receptor of abnormal tau protein seeds.

## Materials and methods

### Antibodies

Anti-tau antibodies used in this study were as follows: T46 (epitope: 395–432; Invitrogen), pS396 (epitope: p-Ser-396; Calbiochem), HT7 (epitope: 159–63; Thermo Scientific), AT8 (epitope: p-Ser-202 and p-Thr-205; Thermo Scientific). Anti-APP antibodies used in this study were as follows: 22C11 (epitope: 66–81; Millipore), R37 (epitope: 756–770, as described [24, 28]).

### Expression and purification of tau protein

An expression plasmid, pRK172, containing human 4R1N tau was expressed in *E. coli* BL21 (DE3). Recombinant 4R1N tau protein was purified as described [17], and dialyzed against 30 mM Tris–HCl, pH 7.5. The dialyzed sample was centrifuged at 113,000×*g* for 20 min at 4 °C and the supernatant was used as recombinant tau monomer. Protein concentration of tau was determined as described [57].

### Preparation of recombinant tau fibrils

Purified recombinant tau (1 mg/mL) and heparin (Acros Organics, 0.1 mg/mL) were incubated at 37 °C in 30 mM Tris–HCl, pH 7.5 containing 10 mM DTT and 0.1 % sodium azide [44]. After incubation for over 1 week, the mixtures were ultracentrifuged at 113,000×*g* for 20 min. The pellet was resuspended in PBS, sonicated using a Titec sonicator, and used as tau fibrils. The protein concentration of the sample was determined.

### Preparation of Sarkosyl-insoluble fraction

Brain samples of 0.5 g from patients with AD (age 80, Braak stage V–VI, occipital lobe) were homogenized in 10 mL of homogenization buffer (HB: 10 mM Tris–HCl, pH 7.5 containing 10 % sucrose, 0.8 M NaCl, 1 mM EGTA). Sarkosyl was added to the homogenates (final concentration: 2 %), which were then incubated for 30 min at 37 °C and centrifuged at 20,000×*g* for 10 min at 25 °C. The supernatant was centrifuged at 100,000×*g* for 20 min at 25 °C. The pellets were further washed with sterile saline and centrifuged at 100,000×*g* for 20 min. The resulting pellets were used as Sarkosyl-insoluble fraction (ppt). This study was approved by the research ethics committee of Tokyo Metropolitan Institute of Medical Science.

### Cell culture, transfection of expression plasmids into cells, and treatment of cells with tau fibrils

Human neuroblastoma SH-SY5Y cells were routinely cultured in Dulbecco’s modified Eagle’s medium (DMEM)/F12 medium (Sigma-Aldrich) supplemented with 10 % (v/v) fetal calf serum, penicillin–streptomycin–glutamine (Gibco), and MEM nonessential amino acids solution (Gibco) in a humidified atmosphere containing 5 % CO_2_ at 37 °C. In this study, SH-SY5Y cells were not neuronally differentiated. For transient expression, the cells were grown to 30–50 % confluence in collagen-coated six-well culture dishes, and transfected with plasmids (1 μg) using FuGENE6 (Roche) according to the manufacturer’s instructions. As tau plasmids, we used human 3R1N, 4R1N, and HA-4R1N tau cDNA in pcDNA3.1 vector. As APP plasmids, we employed human APP-695 (wild-type (WT), F690P, KM670/671NL, V717F, V717G) and APP-C99 cDNA in pEFBOS [38]. APP mutations are indicated as the location of mutation in APP770. Under our conditions, the efficiency of transfection was about 20 %. In treatment of cells with tau fibrils, the culture medium was exchanged at 24 h after transfection of expression vector, and tau fibrils (2 μg) were added. Cells were incubated for 24 h. Then, the medium was exchanged again, and cells were incubated for a further 1–2 days.

### Gel electrophoresis and immunoblotting

The cells were washed with PBS, harvested by centrifugation (1800×*g*, 5 min), and extracted with 150 μL of lysis buffer [50 mM Tris–HCl, pH 7.5, 0.15 M NaCl, 5 mM EDTA, 5 mM EGTA, mixture of protease inhibitors]. The extract was briefly sonicated and ultracentrifuged at 113,000×*g* for 20 min at 4 °C, then the supernatant was collected as a Tris-soluble fraction (TS). The protein concentration was determined by BCA assay. The pellet was solubilized by sonication in 100 μL of lysis buffer containing 1 % Triton X-100 and ultracentrifuged, and the supernatant was collected as a Triton X-100-soluble fraction (TX). The pellet was solubilized in 100 μL of lysis buffer containing 1 % Sarkosyl, then ultracentrifuged, and the supernatant was collected as Sarkosyl-soluble fraction (Sar). The pellet was solubilized in 100 μL of SDS-sample buffer and collected as detergent-insoluble pellet (ppt).

Each sample was separated by 10 % SDS-PAGE, and transferred onto polyvinylidene difluoride membrane (Millipore). The membranes were blocked with 3 % gelatin and incubated overnight with the indicated primary antibody in 10 % calf serum at room temperature. Next, the membranes were washed with PBS and then incubated with a biotin-labeled secondary antibody (Vector) for 1–2 h at room temperature. Signals were detected using an ABC staining kit (Vector). All experiments were performed at least three times, and representative results are shown.

### Confocal immunofluorescence microscopy

SH-SY5Y cells on coverslips were cultured as described above. Then, the cells on the coverslips were fixed with 4 % paraformaldehyde, and permeabilized with 0.2 % Triton X-100 in PBS for 10 min. After blocking for over 30 min in 5 % BSA in PBS, samples were incubated with primary antibody diluted with 5 % BSA in PBS for 1 h at 37 °C. After washing, samples were incubated with Alexa Fluor 488- or 568-labeled goat anti-rabbit or mouse IgG for 1 h at 37 °C. After washing, the samples were further incubated with TO-PRO-3 (Invitrogen) diluted with PBS for 1 h at 37 °C. The cells were analyzed using an LSM780 confocal laser microscope (Carl Zeiss).

### Immunoelectron microscopy

For immunoelectron microscopy, cells overexpressing both HA-4R1N and APP were treated with 4R1N tau fibrils as described above. After incubation, they were harvested and suspended in 1 mL of homogenization buffer containing 1 % Sarkosyl. After incubation for 30 min at room temperature, the homogenates were centrifuged at 113,000×*g* for 20 min at 25 °C. The resulting pellets were suspended in 30 mM Tris–HCl, pH 7.5, and then placed on collodion-coated 300-mesh nickel grids. After drying, the grids were blocked with 30 mM Tris–HCl, pH 7.5 containing 2 mg/mL BSA, and incubated overnight with anti-HA or AT8 antibody at a dilution of 1:200. The grids were rinsed and reacted with secondary antibody conjugated to 10-nm gold particles (1:50), then rinsed again and stained with 2 % (v/v) phosphotungstate. Micrographs were obtained with a JEOL JEM-1400 electron microscope.

## Results

### Tau fibrils bind on cells expressing APP

**Fig. 1:**
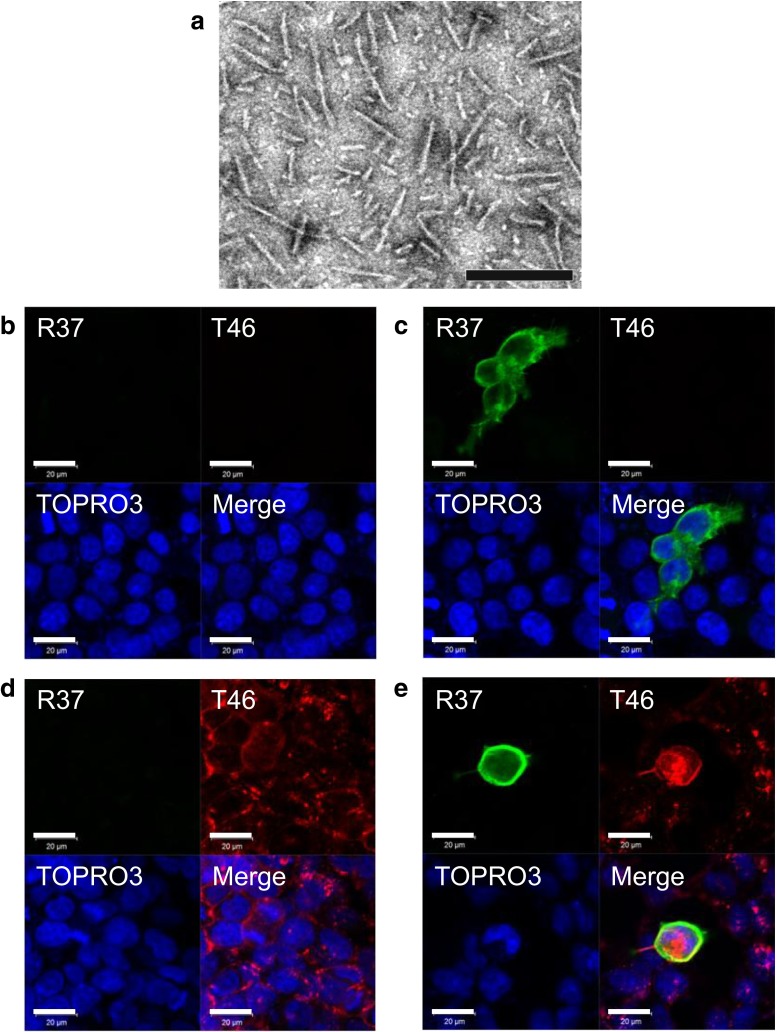
Effect of treatment with monomeric tau or tau fibrils on cells. Electron microscopy of tau fibrils used for the treatment of cells (**a**). The *scale bar* represents 200 nm. Immunohistochemical analysis of cells treated with 4R1N tau monomers (**b**), APP-expressing cells treated with 4R1N tau monomers (**c**), cells treated with 4R1N tau fibrils (**d**), and APP-expressing cells treated with 4R1N tau fibrils (**e**). Cells were immunostained with R37 (APP, *green*) and T46 (tau, *Red*), and counterstained with TO-PRO-3 (*blue*). *Scale bars* represent 20 μm

To explore the putative link between Aβ deposition and tau aggregation, we first examined whether there is any association between APP and tau. We prepared synthetic tau fibrils by incubation of purified tau with heparin, and confirmed fibril formation by means of electron microscopy (Fig. 1a). Next, SH-SY5Y cells were treated with monomeric soluble form of recombinant tau or insoluble tau fibrils for 1 day, then immunostained with anti-tau (T46) and anti-APP (R37) antibodies, and analyzed by confocal laser microscopy. These cells are not transfected with tau, and total treated recombinant tau is detected by T46. Soluble tau was not detected, either in untransfected cells (Fig. 1b) or in cells expressing APP after treatment with monomeric tau (Fig. 1c). In contrast, diffuse T46-positive tau staining was detected on the control cells treated with tau fibrils (Fig. 1d). Furthermore, strong tau staining was observed on APP-expressing cells by incubation with tau fibrils, and this staining was colocalized with APP staining (Fig. 1e). Three representative cross-sectional images of the cells in three dimensions showed that tau fibrils exist both inside and outside of the cells (Fig. S1). These results indicate that fibrillar tau interacts with APP, but soluble tau does not. However, immunoprecipitation experiments using tau or APP antibodies failed to detect the association of tau fibrils with APP, suggesting that the interaction may not be strong (data not shown).

### Exogenously added recombinant tau fibrils induce intracellular aggregation in cells expressing tau and APP

**Fig. 2:**
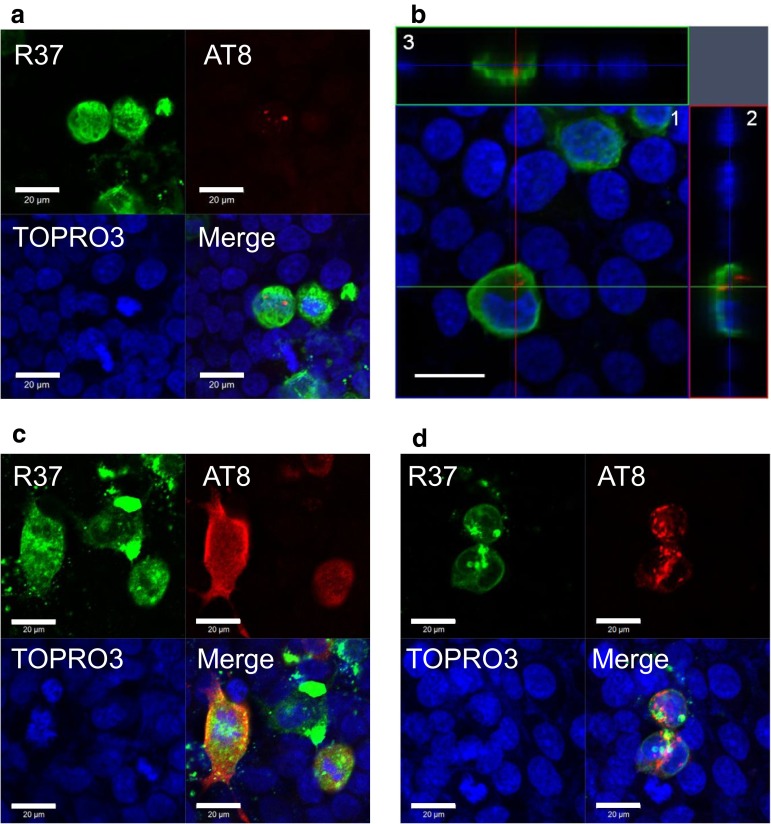
Immunohistochemical analysis of the effect of APP on seed-dependent tau aggregation in cultured cells. Immunohistochemical analysis of APP-expressing cells treated with 4R1N tau fibrils (**a**). Cross-sections of APP-expressing cells after incorporation of tau fibrils (**b**). *1* Optical section (*X*–*Y*) at the depth indicated by the *blue lines* in (*2*) and (*3*). *2* Cross-sectional *Y*–*Z* image along the *green line* shown in (*1*). *3* Cross-sectional *X*–*Z* image along the *red line* shown in (*1*). Immunohistochemical analysis of cells expressing both 4R1N tau and APP, and treated with tau fibrils (**c**, **d**). Cells were immunostained with R37 (APP, *green*) and AT8 (hyperphosphorylated tau, *red*), and counterstained with TO-PRO-3 (*Blue*). *Scale bars* represent 20 μm

It has been reported that tau fibrils are incorporated into cells in the presence of transfection reagent and induce seed-dependent aggregation in tau-expressing cells [42]. In this study, we investigated seed-dependent tau aggregation in the absence of transfection reagent. We examined whether expression of APP affects tau aggregate formation in cultured cells. Cells expressing APP were treated with tau fibrils for 24 h and immunostained with anti-APP antibody R37 and anti-phosphorylated tau antibody AT8, and the cells were analyzed by confocal laser microscopy. AT8 antibody specifically detects phosphorylated tau. Since phosphorylation occurs inside the cells, intracellular tau fibrils can be distinguished from extracellular tau fibrils. In a few APP-expressing cells treated with tau fibrils, small AT8-positive dot-like structures were detected in the cell cytoplasm (Fig. 2a, b). Cross-sectional images of APP-expressing cells with AT8-positive tau in three dimensions showed that the signal of AT8-positive tau is located intracellularly (Fig. 2b). Namely, tau fibrils are incorporated into the cells and then hyperphosphorylated.

Furthermore, when cells expressing both tau and APP were treated with tau fibrils for 24 h, and then for another 24 h after medium change, larger phosphorylated tau inclusions were observed and the frequency of the inclusions in cells was also increased. Two representative results are shown (Fig. 2c, d). Interestingly, these cells showed focal APP immunostaining that was partially colocalized with AT8-positive staining (Fig. 2c). These results indicate that treatment of APP-expressing cells with tau fibrils induces conversion of plasmid-derived soluble tau into hyperphosphorylated and aggregated tau independently of any transfection reagent.

### Biochemical analysis of seed-dependent aggregation of tau

**Fig. 3:**
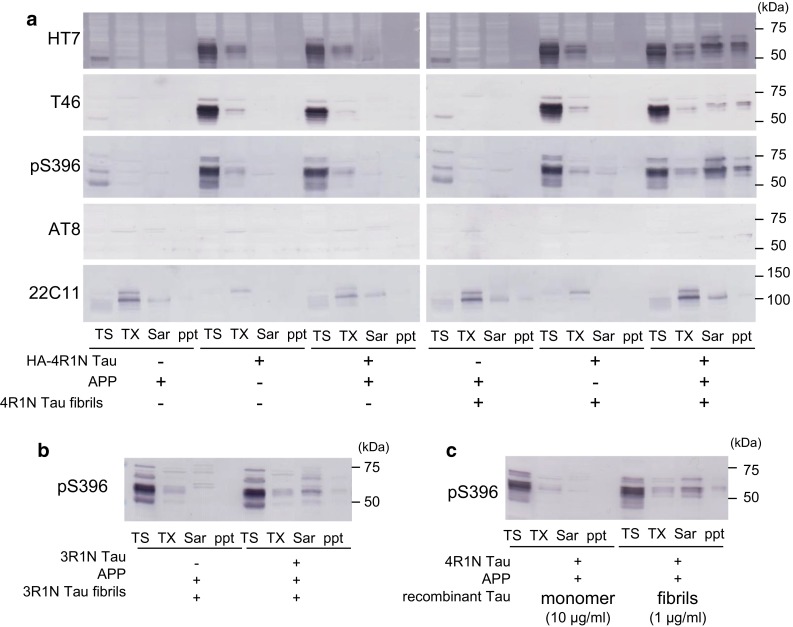
Immunoblot analysis of the effect of APP on seed-dependent tau aggregation in cultured cells. Immunoblot analysis of lysates from cells transfected with APP, cells transfected with 4R1N tau, cells transfected with both 4R1N tau and APP, cells transfected with APP and treated with 4R1N tau fibrils, cells transfected with 4R1N tau and treated with 4R1N tau fibrils, and cells transfected with both 4R1N tau and APP and treated with 4R1N tau fibrils (**a**). Immunoblot analysis of lysates from cells transfected with only APP, and cells transfected with both APP and 3R1N tau and treated with 3R1N tau fibrils (**b**). Immunoblot analysis of lysates from cells transfected with both APP and 4R1N tau and treated with 4R1N tau monomers or 4R1N tau fibrils (**c**). Cells were extracted successively to obtain Tris–HCl-soluble fraction (TS), Triton X-100 soluble fraction (TX), and Sarkosyl-soluble fraction (Sar), leaving the pellet fraction (ppt). Tau was detected with HT7 (159–163), T46 (395–432), pS396 (p-Ser-396), and AT8 (p-Ser-202 and p-Thr-205). APP was detected with 22C11

To confirm the immunocytochemical observations, we performed biochemical analysis. SH-SY5Y cells were transiently transfected overnight with plasmids for expression of 4R1N tau and/or APP, and then the cells were treated with or without tau fibrils (1 μg/mL) for 24 h. After medium exchange, the cells were cultured for another 48 h, harvested and differentially extracted with Tris–Saline (TS), 1 % Triton X-100 (TX), 1 % Sarkosyl (Sar), leaving the pellet fraction (ppt). The fractions were subjected to immunoblot analysis using several anti-tau and anti-APP antibodies. As shown in Fig. 3a, a single faint tau band at 50 kDa was endogenous tau, which was detected in TS fraction with HT7 and T46 in cells transfected with only APP. In cells transfected with 4R1N tau or both APP and tau, a broad tau band was detected at 60 kDa in the TS and TX fractions with HT7, T46 and pS396. However, no immunoreactivity was detected in the Sar-soluble and insoluble fractions (ppt) in these cells, indicating that simple expression of tau and APP cannot elicit intracellular tau aggregation. Similarly, no insoluble tau bands were detected in untransfected cells or in cells transfected with tau, even if the cells were treated with tau fibrils, indicating that extracellular tau fibrils are hardly introduced into cells under these conditions [42]. In contrast, when cells were transfected with both tau and APP, and treated with exogenous tau fibrils, pS396- or AT8-positive hyperphosphorylated tau was detected in the Sar-soluble and insoluble fractions of the cells; thus, APP expression is essential for Sar-insoluble intracellular tau aggregation without transfection reagent. Also, tau has to be overexpressed in the recipient cells, because the amount of endogenous tau is too small to induce aggregation.

Similar results were obtained in cells expressing a 3R tau isoform. An increased amount of Sar-soluble and insoluble 3R tau was detected in cells expressing 3R1N tau and APP when the cells were treated with 3R1N tau fibrils (Fig. 3b). The aggregating ability of 4R tau was essentially the same as that of 3R tau (not shown). Thus, the results clearly show that expression of APP accelerates seed-dependent intracellular tau aggregation in the cells, suggesting that incorporation of extracellular tau fibrils into cells is dependent on the expression of APP.

To confirm that tau fibrils, not monomers, induce seed-dependent intracellular tau aggregation, SH-SY5Y cells expressing both tau and APP were treated with 4R1N tau monomer (10 μg/mL) or fibrils (1 μg/mL) and analyzed by immunoblotting. As shown in Fig. 3c, Sar-soluble and insoluble tau was detected in cells treated with tau fibrils, but no such tau was detected in cells treated with monomeric tau, indicating that only fibrillar tau can induce seed-dependent tau aggregation.

**Fig. 4:**
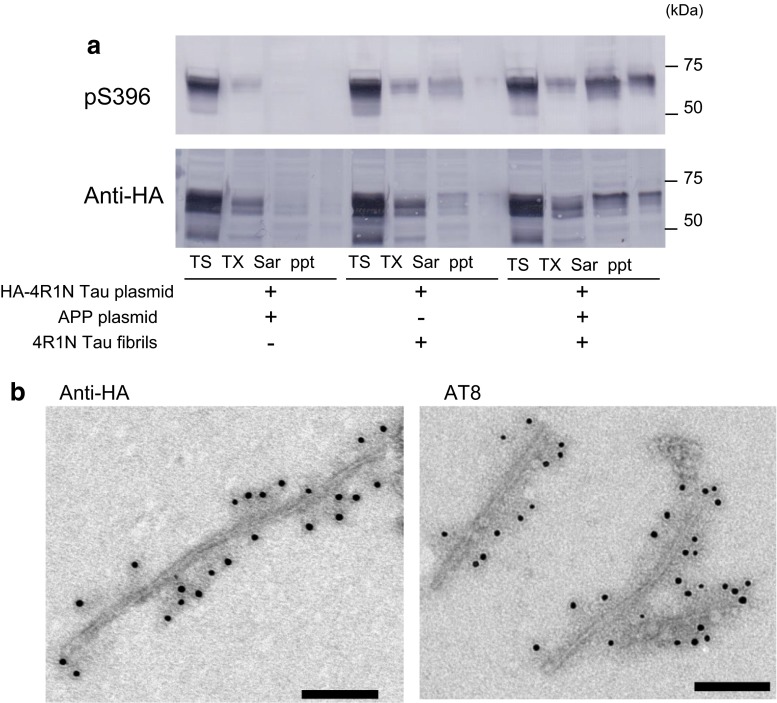
Immunoblotting and immunoelectron microscopic analyses of tau in cells expressing both APP and HA-tagged 4R1N tau. Immunoblot analysis of lysates from cells transfected with both HA-4R1N tau and APP, cells transfected with HA-4R1N tau and treated with 4R1N tau fibrils, and cells transfected with both HA-4R1N tau and APP and treated with 4R1N tau fibrils (**a**). Cells were sequentially extracted to obtain Tris-soluble (TS), Triton X-100-soluble (TX), and Sarkosyl-soluble (Sar) fractions, leaving the pellet fraction (ppt). HA-4R1N tau was detected with pS396 (p-Ser-396) and anti-HA antibodies. Immunoelectron microscopy of tau in the Sar-insoluble fraction from cells transfected with both HA-4R1N tau and APP and treated with 4R1N tau fibrils (**b**). Anti-HA-positive (*left panel*) and AT8 (p-Ser-202 and p-Thr-205)-positive (*right panel*) filaments were observed. *Scale bars* represent 100 nm

To distinguish plasmid-derived intracellular tau from tau fibrils introduced as seeds, we used a plasmid encoding hemagglutinin (HA)-tagged 4R1N tau. SH-SY5Y cells were transiently transfected with both HA-tagged tau and APP, and then transduced with tau fibrils. This treatment also induced accumulation of pS396-positive hyperphosphorylated tau in the Sar-soluble and insoluble fractions, which were labeled with anti-HA antibody (Fig. 4a), indicating that the insoluble tau consists of plasmid-derived exogenous tau. The Sar-insoluble fraction was further analyzed by immunoelectron microscopy, which confirmed accumulation of anti-HA-positive (Fig. 4b right panel) and AT8-positive (Fig. 4b right panel) filamentous tau of 10–15 nm width.

### Tau aggregates from AD brains also accelerate intracellular tau aggregation in APP-expressing cells

**Fig. 5:**
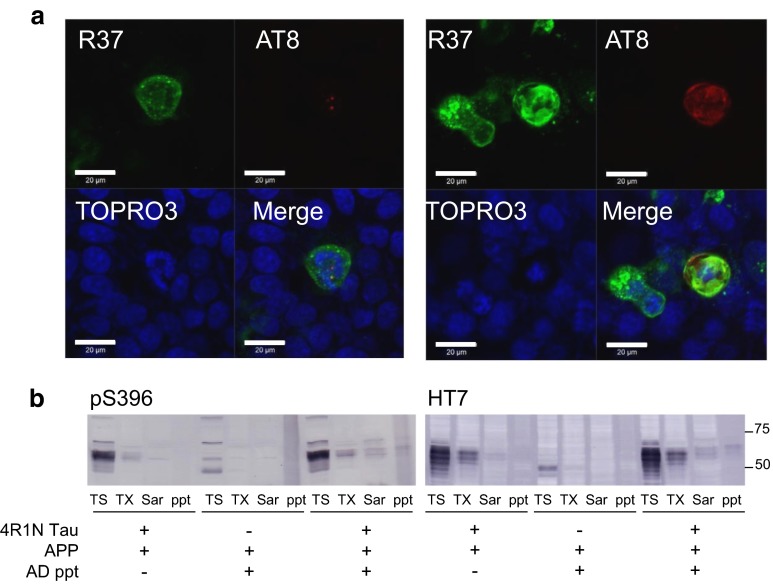
Effect of APP on AD Sarkosyl ppt-dependent 4R1N tau aggregation in cultured cells. Immunohistochemical analysis of cells expressing both 4R1N tau and APP, and treated with AD Sarkosyl ppt (**a**). Cells were immunostained with R37 (APP, *Green*) and AT8 (hyperphosphorylated tau, *Red*), and counterstained with TO-PRO-3 (*Blue*). *Scale bars* represent 20 μm. Immunoblot analysis of lysates from cells transfected with both 4R1N tau and APP, cells transfected with 4R1N tau and treated with AD Sarkosyl ppt, and cells transfected both 4R1N tau and APP and treated with AD Sarkosyl ppt (**b**). Cells were extracted successively to obtain Tris–HCl-soluble fraction (TS), Triton X-100 soluble fraction (TX), and Sarkosyl-soluble fraction (Sar), leaving the pellet fraction (ppt). Detection was done with pS396 or HT7

To examine whether Sar-insoluble fraction prepared from AD brains accelerates the formation of intracellular tau aggregates, SH-SY5Y cells were transfected with both APP and 4R1N tau, followed by incubation with Sar-insoluble fraction prepared from AD brains. Confocal laser microscopy of the cells revealed small AT8-positive dot-like structures or diffusely stained hyperphosphorylated tau in cells expressing APP, which are positive for R37 staining. Two representative results are shown (Fig. 5a). Some of the AT8-positive structures are colocalized with R37, suggesting that tau fibrils, mostly in the form of PHFs, in the Sarkosyl-insoluble fraction of AD brains are incorporated into cells expressing APP.

To confirm this idea biochemically, cells treated with the Sar-insoluble fraction of AD brain were harvested and subjected to immunoblot analysis. As shown in Fig. 5b, Sar-soluble and insoluble tau bands were detected with anti-pS396 and HT7 antibodies, which are associated with expression of APP, although the intensities of these bands were slightly weaker than those of cells treated with recombinant tau fibrils. These results suggest that pathological tau in brains of AD patients also works as seeds for intracellular tau aggregation in cells overexpressing APP even in the absence of any transfection reagent.

### Extracellular domain of APP is required to induce extracellular fibril-dependent intracellular tau aggregation

**Fig. 6:**
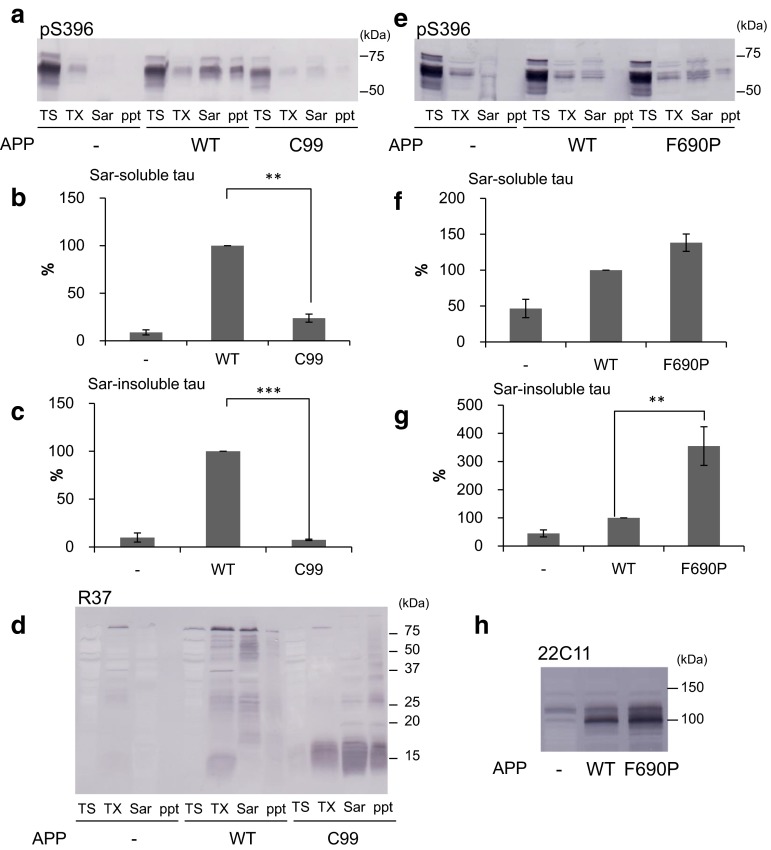
Effect of the extracellular domain of APP on intracellular tau aggregation. Immunoblot analysis of lysates from cells transfected with 4R1N tau, cells transfected with both 4R1N tau and APP, and cells transfected with both 4R1N tau and APP-C99. All cells were treated with 4R1N tau fibrils (**a**). In the *lower panel*, the Sarkosyl-soluble fraction and Sarkosyl-insoluble fraction detected by pS396 are shown. The results are expressed as means +SE (*n* = 3). WT-APP was taken as 100 %. **p < 0.01; ***p < 0.001 by Student’s *t* test against the value of none (**b**, **c**). These cells were also detected using R37 (**d**). Immunoblot analysis of lysates from cells transfected with 4R1N tau, cells transfected with both 4R1N tau and APP, and cells transfected with both 4R1N tau and APP-F690P. All cells were treated with 4R1N tau fibrils (**e**). In the *lower panel*, the Sarkosyl-soluble fraction and Sarkosyl-insoluble fraction detected by pS396 are shown. The results are expressed as means +SE (*n* = 4). WT-APP was taken as 100 %. **p < 0.01 by Student’s *t* test against the value of none (**f**, **g**). The Triton X-100 soluble fractions were detected by 22C11 (**h**)

Given that expression of APP accelerates seed-dependent intracellular tau aggregation, it appears likely that APP play an important role in uptake of extracellular tau fibrils and in aggregate formation of tau in cells. Therefore, we investigated which domain of APP is required for the intracellular tau aggregation. SH-SY5Y cells transfected with both 4R1N tau and wild-type (WT) APP or a truncated form of APP (C99), which lacks the N-terminal extracellular region of APP, were incubated with tau fibrils (1 μg/mL) for 24 h, and cultured for another 48 h after medium exchange. Then, the cells were harvested, fractionated and subjected to immunoblot analysis (Fig. 6a). Quantitative analysis of the immunoblot is in sharp contrast to the strong tau immunoreactivities in the Sar-soluble (Fig. 6b) and insoluble fractions (Fig. 6c) of cells transfected with WT-APP: tau bands were barely detectable in those fractions of cells expressing APP-C99. This result clearly indicates that the extracellular domain of APP is required for seed-dependent intracellular tau aggregation. Expression of WT-APP or of the truncated form C99 was confirmed by immunoblot analysis with R37 antibody (Fig. 6d). WT-APP and APP-C99 were detected at about 100 kDa and 15 kDa, respectively.

Next, we investigated whether the seed-dependent tau aggregation is correlated with the protein levels of APP. The F690P mutation of APP is known to affect the cleavage of APP by α-secretase and to increase the protein levels of APP [16]. Cells expressing both tau and WT-APP or the F690P mutant APP were treated with tau fibrils (1 μg/mL) for 1 h, and cultured for 72 h after medium change. Then, the cells were harvested, fractionated and subjected to immunoblot analysis (Fig. 6e). The Sar-soluble (Fig. 6f) and insoluble (Fig. 6g) tau bands of cells expressing F690P mutant APP were quantitatively more intense than those of cells expressing WT-APP. Expression levels of APP F690P were increased by about 36 % compared with WT-APP (Fig. 6h). This result suggests that elevated expression of APP at the cell membrane may promote the formation of intracellular tau aggregates in these cells.

### Effects of APP pathogenic mutations and Aβ42 on intracellular tau aggregation

**Fig. 7:**
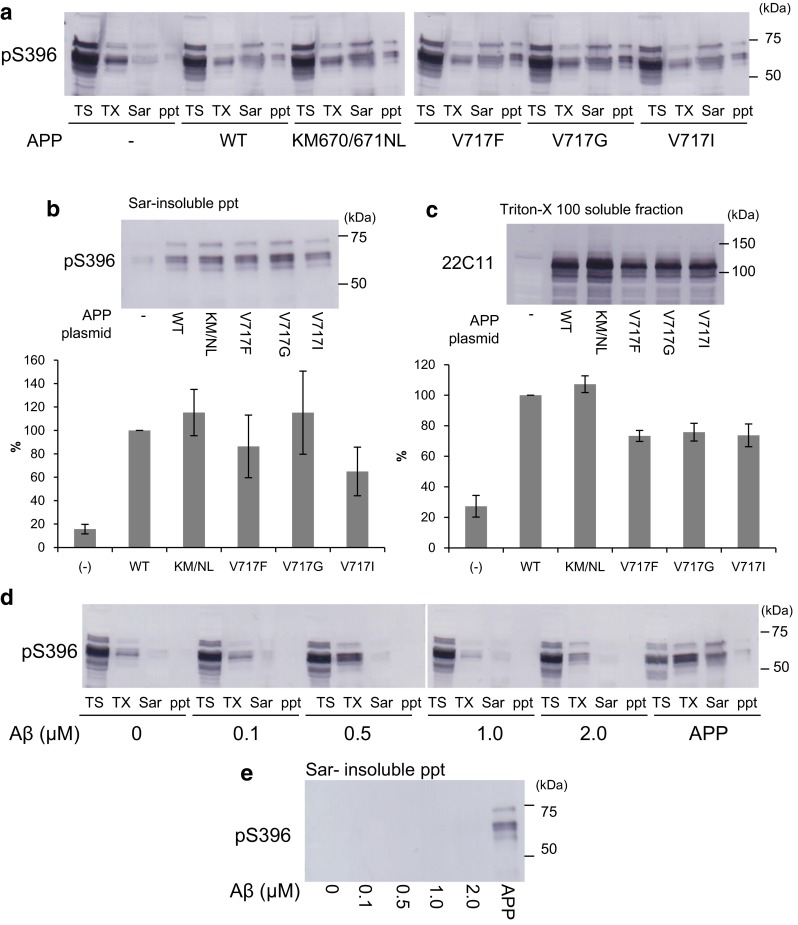
The effect of APP pathogenic mutant and amyloid β on intracellular tau aggregation. Immunoblot analysis of lysates from cells transfected with 4R1N tau, cells transfected with both 4R1N tau and WT-APP, cells transfected with both 4R1N tau and APP KM670/671NL, cells transfected with both 4R1N tau and APP V717F, cells transfected with both 4R1N tau and APP V717G, and cells transfected with both 4R1N tau and APP V717I. All cells were treated with 4R1N tau fibrils (**a**). The Triton X-100 soluble fraction detected by 22C11 (**b**) and the Sarkosyl-insoluble pellet fraction detected by pS396 (**c**) are shown. The results are expressed as means +SE (*n* = 3). WT-APP was taken as 100 %. Immunoblot analysis of lysates from cells transfected with 4R1N tau and treated with amyloid β (0–2 μM), and cells transfected with both 4R1N tau and APP. All cells were treated with 4R1N tau fibrils (**d**). The Sarkosyl-insoluble pellet fraction is shown (**e**). Cells were extracted successively to obtain Tris–HCl-soluble fraction (TS), Triton X-100-soluble fraction (TX), and Sarkosyl-soluble fraction (Sar), leaving the pellet fraction (ppt). Detection was done with pS396

In familial forms of AD, several pathogenic mutations in the APP gene have been identified, and most of these mutants are reported to increase Aβ production or the ratio of Aβ42/40 [4, 11, 40, 41]. Therefore, we investigated whether these APP mutations influence seed-dependent tau aggregation in cultured cells. SH-SY5Y cells expressing WT-APP or a mutant (KM670/671NL, V717F, V717G or V717I) together with 4R1N tau were treated with tau fibrils (1 μg/mL) for 24 h, then cultured for another 48 h after medium change and analyzed by immunoblot analysis. As shown in Fig. 7a, c, expression levels of tau or APP were similar among the samples, and the levels of Sar-soluble and insoluble tau were comparable (Fig. 7b). No significant difference was detected even after normalizing the data for the amount of APP (data not shown), suggesting that these mutations may not affect incorporation of tau fibrils or intracellular tau aggregation. We also analyzed the effect of extracellular Aβ monomer on intracellular tau aggregation. SH-SY5Y cells expressing 4R1N tau were treated with both Aβ42 monomer (0–2 μM) and tau fibrils (1 μg/mL) for 24 h, cultured for another 48 h, and then subjected to immunoblot analysis (Fig. 7d). As a positive control, cells were transfected with expressing 4R1N tau and APP, and treated with tau fibrils. As shown in Fig. 7e, we confirmed accumulation of phosphorylated tau in insoluble fractions of the cells transfected with APP; however, no tau bands were detected in cells treated with Aβ42, clearly indicating that extracellular Aβ42 does not promote intracellular tau aggregation.

## Discussion

In our seed-dependent intracellular aggregation model of full-length tau using full-length tau fibril seeds [42], recombinant tau fibrils were introduced into cells only in the presence of a transfection reagent such as Lipofectamine, as was subsequently confirmed by other groups [14, 61]. On the other hand, Holmes et al. [18] recently reported that heparan sulfate proteoglycans mediate internalization of tau fibrils and promote tau aggregation. However, the mechanism by which aggregated extracellular tau binds to and enters cells to trigger intracellular tau accumulation is still unknown. In the present study, we show that overexpressed APP on the cell surface associates with tau fibrils and accelerates intracellular tau aggregation, and that both recombinant tau fibrils and pathological tau-enriched Sarkosyl-insoluble fraction from AD brains can induce intracellular tau aggregation in association with APP. Furthermore, the extracellular domain of APP is required for the acceleration of seed-dependent tau aggregation.

APP is predominantly cleaved by α-secretase, generating soluble APPα and the corresponding C-terminal fragments (CTF-α) [58]. Intact APP can be internalized from the cell surface by endocytosis, then β- or γ-secretase cleavage can occur intracellularly [25]. The transient expression of APP may increase the activity of cellular endocytosis and metabolism of APP. It is also known that APP has a heparin-binding site in its extracellular region, which is thought to be rich in positively charged amino acids. Recombinant tau fibrils prepared in the presence of heparin or phosphorylated tau aggregates in AD brain might associate electrostatically with the heparin-binding site of APP and be internalized into cells together with APP by endocytosis. Alternatively, the expression of APP may influence cell membrane fluidity, because APP is reported to bind cholesterol [56] and control its turnover [46], so it is possible that aggregated tau may be able to pass through cell membrane with altered fluidity to reach the cytoplasm, where it functions as seeds for intracellular tau aggregation. In any case, extracellular tau fibrils derived from tangle-bearing cells after cell death or released via some cellular secretion system [52, 54] may be incorporated into cells expressing APP, triggering intracellular seed-dependent tau aggregation and subsequent propagation of tau pathology.

Treatment of cells with Aβ42 did not accelerate intracellular tau aggregation in our cellular model. Given that the seed-dependent tau aggregation did not occur in cells expressing mutant APP lacking an extracellular domain, we consider that APP itself rather than Aβ is required for induction of intracellular aggregate formation of tau. Many researchers have proposed a link between extracellular Aβ deposition and intracellular aggregation of tau, and indeed, it has been confirmed that Aβ is associated with tau pathology [3, 7, 22, 33]. However, it is still unclear which protein pathology appears first, and how the two molecules interact with each other. Our present results indicate that APP, but not Aβ, binds tau fibrils or pathological tau and accelerates incorporation of tau and seed-dependent intracellular accumulation of tau. Since we could not detect any significant effect of APP mutations found in rare familial forms of AD on tau aggregation in this study, the pathological relevance of these mutations is unclear. However, it has been shown that development of AD pathology is associated with a 1.5-fold or more increase of expression of APP in Down’s syndrome patients [51, 60] and a twofold increase in patients with APP gene duplication [15, 26, 36, 49, 50, 55]. It is not yet clear whether or not there is any increase in the expression levels of APP in brains of patients with sporadic AD, but it has been reported that the APP expression is increased in brains of post-traumatic injury patients [13, 47, 48] and animal models [21, 30, 43, 45, 59]. It is possible that an increase in the APP following injury or damage to the brain may accelerate tau accumulation and spreading. Further studies are needed to explore the relationship between increase of APP and tau pathologies.

Based on the results obtained in this study, we propose that APP may work as a receptor for uptake of tau aggregates into cells and thereby promote seed-dependent intracellular tau aggregation. Further studies on the role of APP in the pathogenesis of AD and other neurodegenerative diseases using our new cellular model for intracellular tau accumulation in cells expressing APP may lead to the development of new therapies and pharmaceuticals for these diseases.

## Electronic supplementary material

Fig. S1. Immunohistochemical analysis of APP-expressing cells treated with tau fibrils. Cross-sections of APP-expressing cells treated with 4R1N tau fibrils **(a-c)**. (1) Optical section (X–Y) at the depth indicated by the blue lines in (2) and (3). (2) Cross-sectional Y–Z image along the green line shown in (1). (3) Cross-sectional X–Z image along the red line shown in (1). Cells were immunostained with R37 (APP, Green) and T46 (tau, Red), and counterstained with TO-PRO-3 (Blue). Scale bars represent 20 μm (PDF 261 kb)
