# Supplementary figures and images for: Extracellular association of APP and tau fibrils induces intracellular aggregate formation of tau
Source: Acta Neuropathol. 2015 Apr 14;129(6):895–907. doi: 10.1007/s00401-015-1415-2 (PMC4436700; doi:10.1007/s00401-015-1415-2)

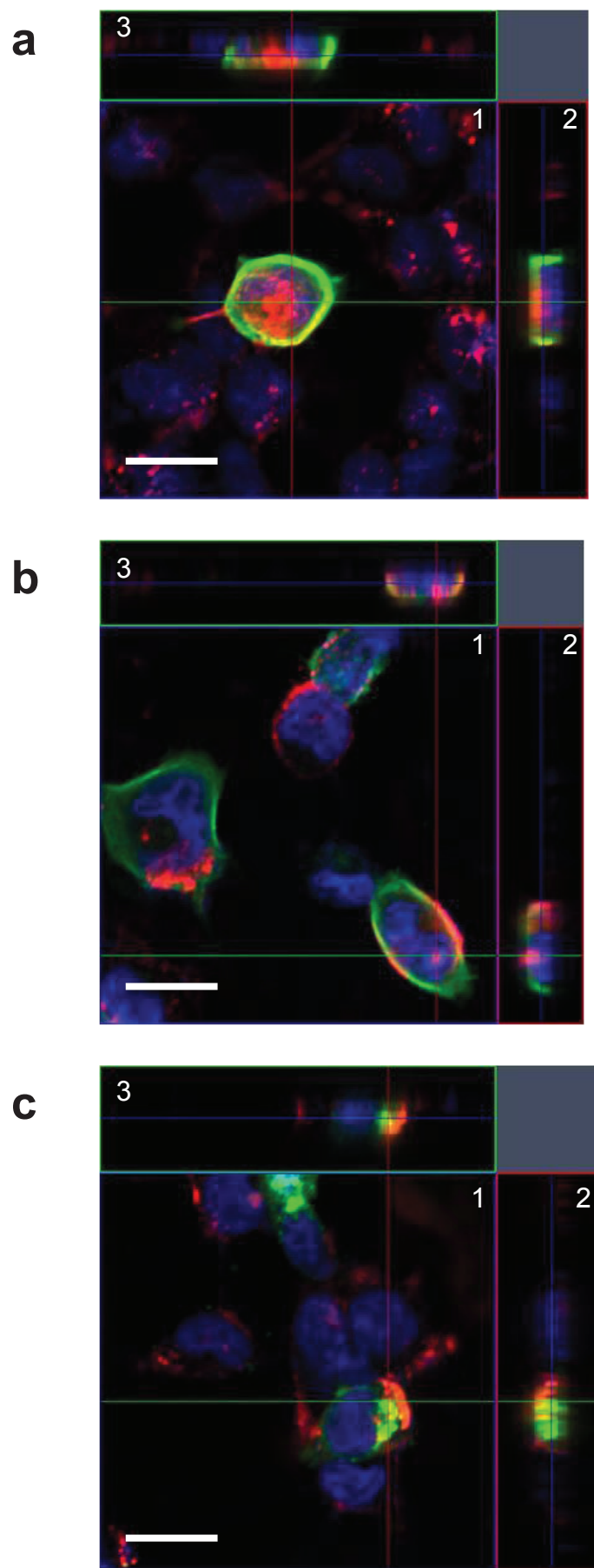

**Fig. S1**

Supplement: Supplementary file 1 — Fig. S1. Immunohistochemical analysis of APP-expressing cells treated with tau fibrils. Cross-sections of APP-expressing cells treated with 4R1N tau fibrils (a-c). (1) Optical section (X–Y) at the depth indicated by the blue lines in (2) and (3). (2) Cross-sectional Y–Z image along the green line shown in (1). (3) Cross-sectional X–Z image along the red line shown in (1). Cells were immunostained with R37 (APP, Green) and T46 (tau, Red), and counterstained with TO-PRO-3 (Blue). Scale bars represent 20 μm (PDF 261 kb) [file 401_2015_1415_MOESM1_ESM.pdf]
